# Dyslexia on a continuum: A complex network approach

**DOI:** 10.1371/journal.pone.0208923

**Published:** 2018-12-17

**Authors:** Erica S. Edwards, Kali Burke, James R. Booth, Chris McNorgan

**Affiliations:** 1 Department of Psychology, State University of New York at Buffalo, Buffalo, New York, United States of America; 2 Department of Psychology and Human Development, Vanderbilt University, Nashville, Tennessee, United States of America; University of Texas at Austin, UNITED STATES

## Abstract

We investigated the efficacy of graph-theoretic metrics of task-related functional brain connectivity in predicting reading difficulty and explored the hypothesis that task conditions emphasizing audiovisual integration would be especially diagnostic of reading difficulty. An fMRI study was conducted in which 24 children (8 to 14 years old) who were previously diagnosed with dyslexia completed a rhyming judgment task under three presentation modality conditions. Regression analyses found that characteristic connectivity metrics of the reading network showed a presentation modality dependent relationship with reading difficulty: Children with more segregated reading networks and those that used fewer of the available connections were those with the least severe reading difficulty. These results are consistent with the hypothesis that a lack of coordinated processing between the neural regions involved in phonological and orthographic processing contributes towards reading difficulty.

## Introduction

Reading is a multisensory task in which orthographic representations (letters or graphemes) are decoded into their associated speech sounds (phonemes). Learning to read entails learning to map between graphemes and phonemes. Some languages differ in the degree to which letters or other graphemic structures directly relate to a single phoneme [1–3]; referred to as the transparency of the language. The English language is categorized as an opaque language as multiple sounds can be represented with a single graphemic structure [1,4]. Thus, the opaqueness of the English language presents us with a prime opportunity to study reading difficulty due to the inconsistent orthography-to-phonology mapping.

One way to learn how the human brain decodes written language is through studying individuals who struggle with reading. One of the most common learning disabilities is dyslexia affecting over 80% of those diagnosed with a learning disability [5]. Several studies have shown that children with dyslexia have difficulties with the audiovisual integration of information required in reading [6–10]. For example, McNorgan, Randazzo-Wagner [11] showed that dyslexic children have an audiovisual disconnect compared to their matched typically-developing counterparts, suggesting that task conditions with higher audiovisual integration demands may be sensitive to reading difficulty.

### The reading network

Much of the research done with dyslexic readers has attempted to identify a neurobiological phenotype for dyslexia through the use of neuroimaging techniques. These neuroimaging methods have helped inform researchers about how the brain processes written language. Reading-related audiovisual processing is broadly supported by four cortical regions, which will be referred to as the reading network in this paper: The fusiform gyrus (FG), which is involved in processing orthographic information [12–14], the posterior superior temporal gyrus (pSTG), which is involved in processing phonology [8, 10, 15–18], the posterior superior temporal sulcus (pSTS), which is involved in cross-modal integration of visual and auditory information [12, 19–21], and the inferior frontal gyrus (IFG), which has been associated with later high-level phonological recoding while reading [10, 22–24]. Because the reading network is dominantly left-lateralized, our study focuses on the reading network within the left hemisphere.

The left hemisphere reading network includes a number of functionally-specialized regions that jointly contribute to normal reading. Within the fusiform gyrus can be found the putative visual word form area (VWFA), so-named because some argue that it is specialized for processing written language [8, 13, 14]. The FG becomes more responsive to word like stimuli over the course of reading development; however, individuals with dyslexia show under-activation in this region during word reading tasks [12, 13, 25]. The posterior superior temporal gyrus is linked to basic phonological decoding [8, 15–18]. In typically developing readers, activation in the pSTG increases during rhyming judgment tasks [18], whereas dyslexics have been shown to have an under-activation in this region [26–28]. The posterior superior temporal sulcus has been implicated in specialized cross-modal integration necessary for reading [18, 20, 21]. Activation in the pSTS increases in cross-modal conditions [19, 29, 30]. Less activity in the pSTS has been associated with lower success in linking letters to their appropriate sounds [12]. The inferior frontal gyrus is associated with speech-articulatory phonological recoding, even in silent reading [10, 15], and contains the language-critical region, Broca’s Area. Further, greater activation in the IFG is associated with less familiar and irregular words [22, 24, 31], suggesting that the IFG is sensitive to spelling-sound irregularities. In summary, the four critical nodes in the reading network on which we focus—the fusiform gyrus, posterior superior temporal and inferior frontal cortices—contribute holistically towards the process of mapping visual to phonological representations in normal reading.

### Brain connectivity within the reading network

These anatomically distributed brain regions each support different reading-critical processes, and the overall reading process must coordinate and integrate each of these otherwise independent processes. Studies of the neuronal mechanisms underlying reading have progressed over time from the earlier studies cited above that identified the neural subpopulations (and by assumption the cognitive processes) that critically support reading, to more recent studies that explore how these regions connect and interact with one another. These studies suggest a model of reading difficulty as a consequence of disordered communication among regions involved in relaying and integrating audiovisual information [11]. Quantitative analyses of brain connectivity are enabled by graph-theoretic metrics that numerically summarize network connectivity patterns [32]. Brain connectivity can be described in terms of structural and functional connections: Structural connectivity describes physical anatomical connections in a network (e.g., white matter projections linking cortical and subcortical regions), whereas functional connectivity refers to the statistical patterns among regions within the network generally derived from measurements of brain activity over time. Functional connectivity between two regions is often inferred from statistical correlations between time series of regional activity. The present study examines how reading difficulty relates to the dynamic functional connections that emerge throughout the reading network under different audiovisual loads.

Mathematical graphs are collections of nodes connected by edges. In our context, nodes refer to discrete cortical regions, and edges refer to the connections, or the probable mutual influence between any two nodes. The numerical value assigned to an edge or connection is an indicator of the existence of (1 or 0 if binary) or strength of the connection (-1.0 to 1.0 if non-binarized correlations) between any pair of nodes. A complete description of connectivity strength between all possible pairs of network nodes can be represented in an adjacency matrix for that network. Though connectivity strength metrics are useful for investigating whether specific functional connections play important roles in reading, graph-theoretic approaches additionally provide a means of quantifying the manner in which a network is connected. This allows us to ask whether patterns of connectivity—in addition to the strength of connectivity—is an important determinant of reading ability.

**Fig 1:**
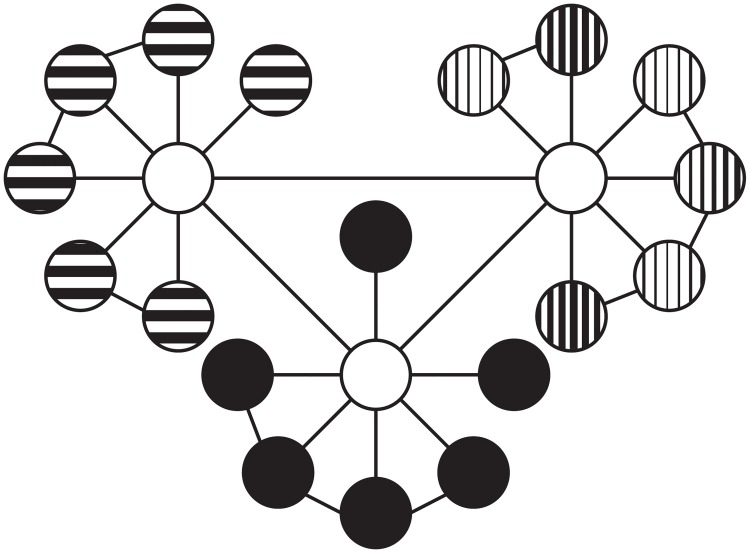
Modularity quantifies a network’s tendency to partition into segregated cliques. Nodes within this network fall into clearly separate groups with few edges between them. This network is high in modularity.

**Fig 2:**
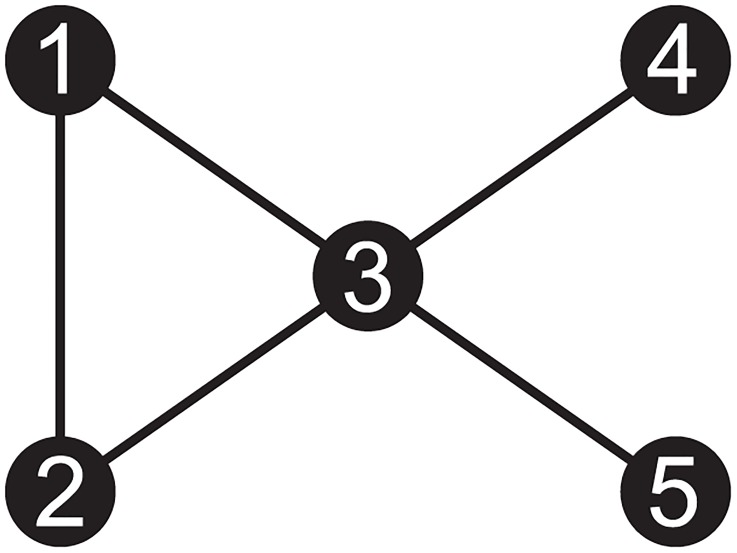
Transitivity quantifies a network’s mutual interconnectedness. Transitivity is computed as the ratio of triangles to triplets. Nodes 1–3 form a triangle while nodes 1,3,5; 2,3,4; & 3,4,5 form triplets.

Patterns of network connectivity can be summarized in terms of segregation and integration. Segregation measures examine the degree to which nodes cohere into clusters, which may signal functionally specialized information processing. Important measures of segregation include modularity and transitivity. Modularity quantifies this characteristic as the degree to which a network may be divided into clearly separate groups (Fig 1), with larger positive values of modularity signifying stronger clustering patterns within a network [33]. Because reading entails multiple processes operating over different types of information (e.g., acoustic and visual representations), each of which likely requiring somewhat specialized neural circuitry, measures of segregation allow the quantification of the number and extent of any such circuits. Mathematically, transitivity is computed as the ratio of triangles to triplets [34]. When two nodes are connected to the same node, they form a triplet; however, when all three nodes are connected, this triplet forms a triangle. In a collection of nodes with high-transitivity, most of the triplets are triangles, indicating that the collection is densely interconnected, and that they form a cluster (Fig 2). One potentially interesting property of a triangular configuration, from an information processing perspective is that they form a cycle, or a recurrent loop, in which the output of previous processing can feed back and influence subsequent processing, and provides the foundational architecture for a memory circuit in neural network architectures [35]. A network may contain several clusters (i.e., collections of nodes with dense interconnections among themselves), but, the nodes within these clusters may have very few connections to nodes that are not part of the cluster.

**Fig 3:**
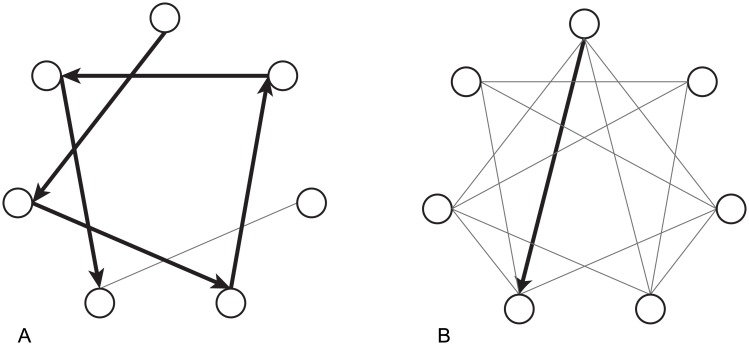
Global efficiency indexes average ease of transmission between nodes. A network with few connections between nodes is low in global efficiency as it takes many jumps to transfer information from one node to another (A). A network with many connections between nodes is high in global efficiency as it takes fewer connections to transfer information from one node to another (B).

Measures of network integration index a networks capacity to exchange information among its nodes. A key measure of network integration is global efficiency, computed as the average inverse shortest path length [32]. A path is any unique pattern of links from one node to another, and its length is equal to the number of steps (in a binary network) or the sum of the links lengths (in a weighted network). In a network with low global efficiency, passing information between two regions requires the signal to pass over a long and circuitous route (Fig 3A). Higher global efficiency is a sign that most network clusters have short (i.e., direct or strong) connections to many other clusters (Fig 3B). Importantly, there may be contexts in which the direct relay of information between two nodes may be suboptimal, as when the nodes participating in a longer circuit perform useful or specialized computations on or transformations of a signal.

**Fig 4:**
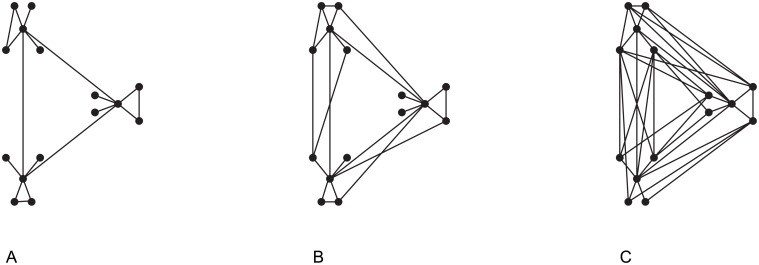
There is often a tension between network segregation and integration. The nodes in many networks naturally fall into clusters, or modules (A). Identifying clusters increases in difficulty as transitivity and global efficiency increase; however, the tension between network segregation and integration is resolved through small world organization (B). As network integration increases discrete clusters decrease (C).

Segregation and integration can exhibit a dependent relationship. In a highly modular network, efficiency decreases as signals must propagate over more steps to traverse the network (Fig 4A). In contrast, a network with high global efficiency may have high transitivity, but no longer have distinct modules (Fig 4C). The tension between segregation and integration is resolved in the ubiquitous small-world organization [32], which reach a compromise between segregation and integration values (Fig 4B).

### Network analytic approaches to studying reading difficulty

Much research has been directed at identifying and ascribing functionality to the neural structures supporting reading-related processes, but work has more recently shifted focus to the functional connectivity within and between these regions, as these studies critically inform us how these regions dynamically interact during normal and impaired reading. Wise Younger, Tucker-Drob [36] suggest that not only is dyslexia defined by under-activation of the brain regions associated with reading, but also reduced functional connectivity between these regions. Others have supported this finding, showing that the integrity of connections within the brain predict reading skills [20, 37–49]. Collectively, these studies suggest that reading skill is dependent on the strength of inter-regional connectivity within the reading network.

Strength, however, is only one aspect of connectivity, and as studies of small-world networks have shown, network configuration plays an important role in how information is communicated among connected nodes [32]. Consistent with this perspective are studies showing that reading development is predicted by changes in how brain regions interact [50–54]. For instance, one study showed that better adult reading performance was related to stronger functional connectivity between the visual word form area and regions associated with phonological processing, whereas poorer reading skills in children were predicted by negative associations in these connections [54]. Moreover, connectivity between the IFG and posterior task-selective regions was weaker in children compared to adults when completing rhyming and spelling judgment tasks [53]. Converging evidence suggests that as reading becomes more automated reliance shifts from phonological specific regions to visuo-orthographic regions; however, stronger connectivity between phonological processing units and higher-level cognitive control regions is associated with better rhyming judgment in both groups [55]. This shift from a reliance on phonology to a reliance of orthography appears to be slower in dyslexic readers [36, 37]. However, Horowitz-Kraus and colleagues [56] showed that after participating in a successful reading intervention program, Reading Acceleration Program, children who experience reading difficulty had similar functional connectivity to their non-impaired reading counterparts.

Much of the connectivity work investigating differences between non-impaired and dyslexic readers has been conducted on resting state data. Though graph-theoretic studies of intrinsic network connectivity often make use of very large resting state data sets, such as the functional connectomes project [57], important insights into specific cognitive processes can be gained when processing dynamics are constrained by tasks that are representative of the processes in question. Further, Gonzalez-Castillo and Bandettini [58] claim that functional connectivity analyses on task-based data are essential to gaining a better understanding of the relationship between functional connectivity for resting-state and task-based data. For example, Vogel and colleagues [59] found that reading related brain regions perform other general tasks and are not necessarily co-activated with one another outside of reading. We thus explore how reading skill depends on connectivity characteristics among these regions using data collected during a reading task. The multisensory nature of reading and its dependency on audiovisual integration implies that the task used in our study should be sensitive to atypically developing connectivity among the brain regions supporting auditory, visual, and integrative processing. McNorgan, Randazzo-Wagner [11] identified differences between typically developing readers and those with reading difficulty with respect to orthographic/phonological congruency processing under unimodal auditory and cross-modal audiovisual presentation conditions. Children with reading difficulty showed no relationship between brain activity and phonemic awareness under any modality condition, whereas typically developing children showed a relationship under the cross-modal condition. Horowitz-Kraus, Buck [60] showed that children with reading difficulties also significantly differ in their auditory comprehension compared to non-impaired readers and this relationship is associated with global efficiency, such that higher global efficiency predicted poorer narrative comprehension. Both of these findings support the position that reading difficulty was attributable to a disconnect between orthographic and phonological processing, and suggests that the connectivity dynamics under the audiovisual condition might be most sensitive to reading difficulty. Thus, we hypothesize that the severity of reading difficulty may be explained by differences in functional brain connectivity. The ability to examine the connectivity dynamics of the reading network under different presentation modality conditions further afforded the opportunity to investigate whether changes to information flow induced by different audiovisual integration demands may be differentially sensitive to reading difficulty.

## Methods

### Participants

Twenty-four children with dyslexia (*M* = 10 years; 9 months old, range: 7 years; 10 months to 13 years; 8 months old; 15 males, 9 females) were recruited from the Chicago metropolitan area. All children had been previously assigned a dyslexia diagnosis by a qualified professional, which we further corroborated with the battery of standardized tests that were administered to all prospective participants (Table 1). Parents were interviewed to confirm that all participants also met the following criteria: (1) native English speaker, (2) right-handedness, (3) free of neurological disease or psychiatric disorders, and (4) no attention deficit hyperactivity disorder (ADHD). The informed consent and all data collection and archival procedures for this study were approved by the Institutional Review Board at Northwestern University.

**Table 1:** Mean scaled scores and standard deviations (in parentheses) for standardized tests of achievement.

Standardized Measure	M (SD)	Range
Full Scale IQ (WASI)	103.1 (15.9)	79–136
Word Identification (WJ III)	88.6 (6.2)	76–101
Word Attack (WJ III)	93.3 (6.4)	78–104
Reading Fluency (WJ III)	88.1 (8.0)	67–110
Sight Word Efficiency (TOWRE)	91.8 (9.2)	67–113
Pseudo-word Decoding Efficiency (TOWRE)	90.5 (9.3)	65–104

After parents gave written informed consent, the children were assessed with a series of standardized tests as an initial participant prescreening measure. Two verbal subtests (vocabulary and similarities) and two performance subtests (block design and matrix reasoning) from the Wechsler Abbreviated Scale of Intelligence (WASI) [61] were used to measure intelligence. All participants’ full scale IQ scores were above 75. The Word Identification, Word Attack, and Reading Fluency subsets of the Woodcock Johnson III Tests of Achievement (WJ-III) [62] assessed word and non-word reading accuracy. Reading speed for words and non-words was measured with the Test of Word Reading Sight Word Efficiency (TOWRE-SWE) and the Pseudo-word Decoding Efficiency (TOWRE-PDE) subtests, respectively [63]. The TOWRE subtests measure the number of consecutively presented written items that individuals can pronounce. The TOWRE subtests quantify reading efficiency by counting the number of written items an individual can pronounce aloud within 45 seconds. The difficulty level of the TOWRE items increases as one progresses through the list. The scores from the TOWRE-PDE were used as our dependent measure of single-word decoding skill for two reasons. First, this task requires access to letter-sound mapping rules in order to pronounce the stimuli, and prevents using basic visual memory of known words as an aid to word identification. Second, past and ongoing studies of typically developing children [9, 64] have found that developmental changes in PDE scores are predicted by changes in connectivity over the same period. Because the classification of reading and other learning difficulties may be the subject of disagreement, the remaining standardized measures provide quantifiable support for our participants as a sample of children with reading difficulty.

Participants completed a practice session of the experimental task with a set of stimuli not used in the actual experiment in a scanner simulator. Within a week of the simulated scanner session, participants visited the MRI scanner to complete the experimental session. Following a structural (T1-weighted) MRI scan, functional data were acquired. The list order was optimized for an event-related design using OptSeq (http://surfer.nmr.mgh.harvard.edu/optseq), and was fixed for all subjects.

### Experimental procedure

#### Rhyming task

**Fig 5:**
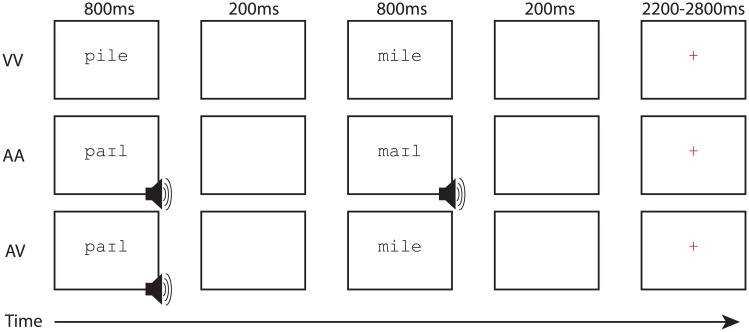
Experimental paradigm. The diagram shows the within-trial event onsets and response window for the VV unimodal task condition, AA unimodal task condition, and AV cross-modal task condition.

Word pairs were presented in a fixed order to the participants and they were asked to indicate if each pair rhymed by pressing one button with their right index finger for ‘yes’ responses, and pressing a different button with their right middle finger for ‘no’ responses. The presentation modality for each word was crossed, creating three presentation modality conditions: both words presented visually (VV); both words presented audibly (AA); and the first word audibly and the second word visually (AV). Visually-presented words appeared on the screen for 800 ms followed by a 200 ms blank interval. Audibly-presented words followed the same timing, though pronunciation times varied slightly. A red fixation appeared on the screen after the second word to indicate that the participant needed to make a response. Responses made prior to the onset of the red fixation cross were ignored, and thus the response window for all presentation modality conditions was the same. A participant response triggered the disappearance of the red fixation cross until the end of the trial, after which time participants viewed a blank screen for the remainder of the jittered inter-trial interval lasting between 2200 and 2800 ms to allow for deconvolution of the signal associated with each condition. Participants completed two runs for each presentation modality condition, each lasting approximately 6:45 minutes. All words and symbols (see Fig 5 below) were presented in lower case, at the center of the screen, with a .5 letter offset of position between the first and second stimulus.

Two levels each of orthographic and phonologic similarity were crossed, creating four different trial conditions: congruent orthography and phonology (O+ P+; e.g., gate, hate), congruent orthography and incongruent phonology (O+ P-; e.g., pint, mint), incongruent orthography and congruent phonology (O- P+; e.g., has, jazz), and incongruent orthography and phonology (O- P-; e.g., press, list). There were 24 trials of each lexical condition, totaling 96 separate word pairs. The same 96 word pairs were used in all three modality conditions. The four trial types were matched for their written word frequency based on child norms [65]. All words were monosyllabic and did not have homophones or homographs.

### MRI data acquisition

Imaging data were obtained using a standard head coil at a 3.0 Tesla Siemens scanner. In order to minimize movements foam pads were used to secure the children’s heads in place. Through a mirror attached to the inside of the head coil, participants were able to view the stimuli projected on a screen. An optical response box was used to record participants’ responses. The echo planar imaging (EPI) method was used during the task to acquire blood oxygenation level dependent (BOLD) images. The following parameters were used for scanning: TE = 20 ms, flip angle = 80°, matrix size = 128 x 120, field of view = 220 x 206.25 mm, slice thickness = 3 mm (0.48 mm gap), number of slices = 32, TR = 2000 ms, voxel size = 1.72 mm. In addition, structural T1 weighted 3D images were acquired (MPRAGE, TR = 1570 ms, TE = 3.36 ms, flip angle = 20°, matrix size = 256 × 256, field of view = 240 mm, slice thickness = 1 mm, number of slices = 160, voxel size = 1 mm x 1 mm).

#### Image analysis

Functional MRI data preprocessing and General Linear Model analyses were performed using the FreeSurfer 5.3.0 Functional Analysis Stream (FSFast) (https://surfer.nmr.mgh.harvard.edu/). This surface-based analysis maps cortical gray matter voxels to a 3-dimensional mesh tessellation of each participant’s structural MRI. Surface meshes for all participants share a common coordinate system that permit co-registration of spatial locations between brains of different shapes and sizes. After each participant’s T1 volume was mapped to a tessellated surface mesh, the EPI volumes were co-registered to the T1 volume, and a 6-parameter rigid body motion correction was applied, and the movement parameters saved as nuisance regressors for the subsequent analysis. The motion corrected EPI volumes were processed in the FreeSurfer template average surface space using a 6 mm full width half maximum smoothing kernel and slice time correction.

#### Region of interest definitions

**Fig 6:**
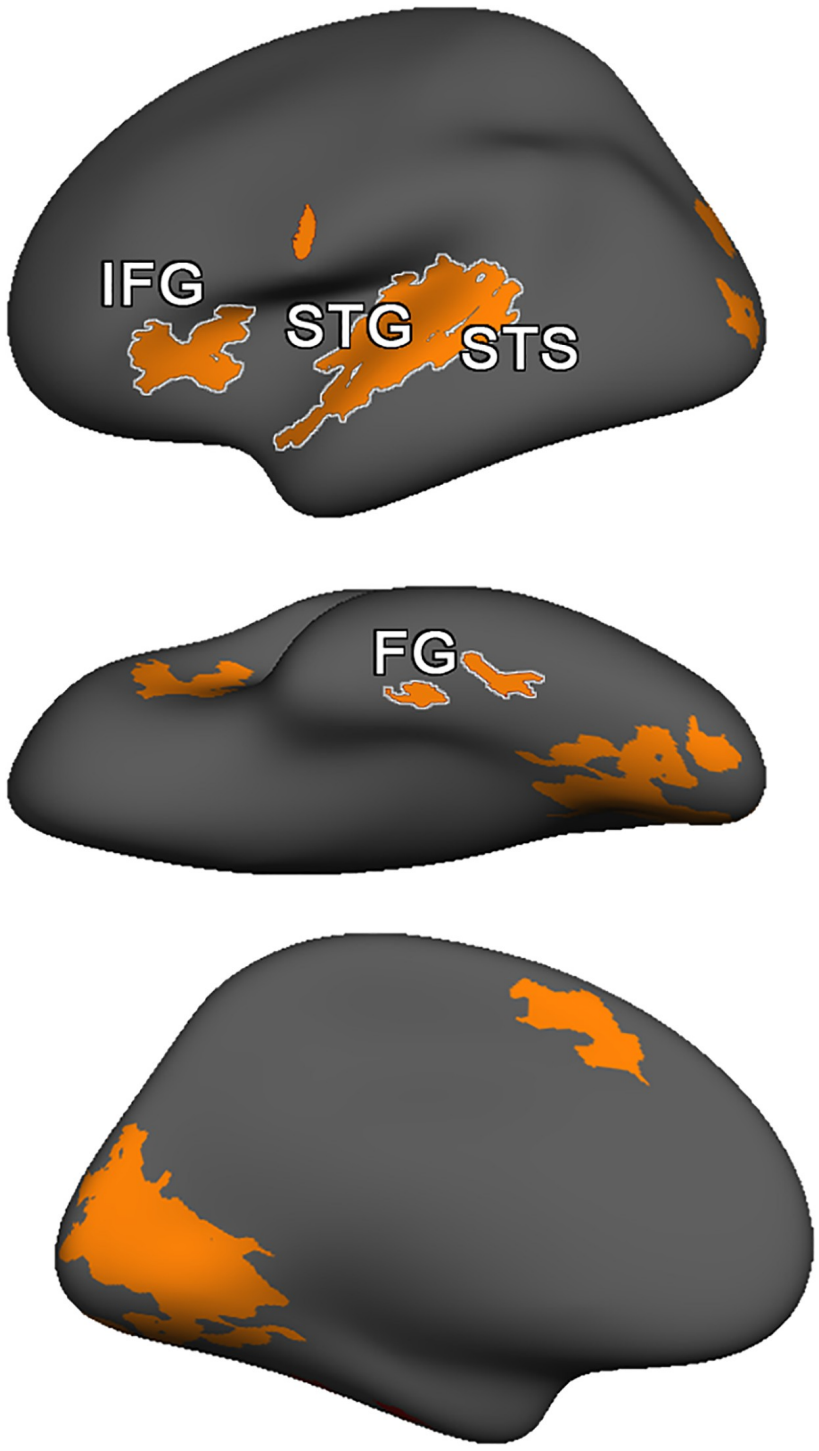
The reading network as defined by a group-level lexical vs. baseline contrast. Significant FWE-corrected (p < .05) clusters in the random-effects lexical vs. fixation baseline contrast, collapsed across all presentation modality conditions functionally define the reading network for the functional connectivity analysis. This definition ensures the network analyses focused on brain regions that were generally engaged in reading-related processing in all presentation modality conditions and across all participants.

We defined our cortical regions of interest both anatomically and functionally in two steps. First, automated anatomical labeling of the cortical surface was constructed based on the Desikan-Killiany atlas [66], as is routine in FreeSurfer’s surface mapping procedure. Each atlas label was subdivided into roughly-equal-sized sub-regions, under the assumption that the neural populations contained within smaller subdivisions are likely more homogeneous than are the populations of larger-scale anatomical features, such as the STG. This parcellated the cortical surface in to 1,000 sub-regions, each of which covered an average of 149 mm^2^, and corresponded to a node in our graph-theoretic analysis. As a consequence, larger anatomical regions (e.g., STG) were subdivided into more nodes than were smaller regions (e.g., FG), however individual nodes were comparable in size. Through FreeSurfer’s surface tesselation and labeling procedure, each voxel within the 3-dimensional MRI volume is assigned an anatomical label based on its co-registration with the template surface mesh. In the second step, significant activation clusters in group-level functional contrast map was intersected with the labeled anatomical map to identify regions on the cortical surface that were associated with the experimental task. The functional contrast map used a first-level one-sample t-test contrasting all lexical trials vs. fixation to identify voxels that were significantly more active during reading than baseline. A second-level (group) random effects analysis selected clusters with a FWE of *p* < .05 containing voxels reaching an uncorrected significance threshold of *p* < .001 to functionally define the left hemisphere reading network, illustrated in Fig 6. From among the nodes generated by the first step that intersected with the significant clusters revealed in the functionally defined reading network, we restricted our analyses to the 43 nodes contained in the four anatomical regions described earlier as comprising the core reading network (i.e. FG, STG, STS and IFG). These 43 nodes were used in the subsequent complex network analysis. Though some related studies in this population have additionally implicated Inferior Parietal Lobule in letter-sound mapping [25, 67], this region did not intersect with the functionally-defined clusters revealed in the random-effects analysis, and thus were not included in our reading network definition but was included in analyses of regions outside the core reading network. A second group level analysis, contrasting left-hemisphere activation for each presentation modality condition against the others was performed to provide a context to facilitate the interpretation of the network analysis.

### Planned connectivity analyses

For each participant, normalized fMRI time series were extracted from each of the nodes comprising the left hemisphere reading network. This was done for each of the six fMRI runs (two runs for each of three presentation modality conditions). For each of these modality conditions, zero-lag cross-correlations were computed between all pairs of time series vectors to produce two 43×43 adjacency matrices (one for each run) for each presentation modality. Because the experiment used a jittered fast event-related design, we limited our analysis to zero-lag correlations. Though this prevented the detection and analysis of lag-dependent processing dynamics across the network, we made this decision to reduce the complexity of the analysis and the uncertainty surrounding inferences about lag-dependent differences that may be influenced by varied event onset schedules for different trial types.

Within adjacency matrices constructed in this manner, all nodes are connected to one another to some degree, necessitating a thresholding step to differentiate the binarized networks from one another. Multiple approaches have been proposed for thresholding to connectivity matrices [68], and the arbitrarily selected threshold is likely to impact the results of comparisons between networks. Homogeneously densely connected networks are likely to result from extremely lax thresholds; homogenously disconnected networks are likely to result from extremely stringent thresholds, and condition differences are unlikely to be detected in either case. Using intermediate thresholds, systematic relationships between experimental condition and connection strength could plausibly allow threshold selection to dictate the directionality of contrasts between connectivity metrics associated with these conditions. One advantage of our regression-based analysis, however, is that we do not directly compare the metrics that quantify network topography. Rather, our regression-based analyses instead ask whether these metrics are predictive of reading skill across individuals, and are dependent on participant variability among connectivity metrics, which cannot be prescribed by threshold selection. We wished to make as few assumptions as possible when thresholding our networks, and so we used two very different but straightforward approaches. One thresholding approach applied a simple significance threshold to include only significant correlations (*p* < .05, not corrected for multiple tests), with all other values set to zero to producing a binary connectivity matrix, **M**, for each participant, where each entry **M**_i,j_ = (0,1) indicates the presence or absence of a connection between nodes i and j during a particular presentation modality condition. The second thresholding technique used the minimum connected component (MCC) of each adjacency matrix. The MCC of a network is graph-theoretic construct in which a subgraph is built by iteratively connecting nodes with the strongest weights in descending order until all the original nodes have been connected. This approach has several advantages: First, it eliminates the need for the experimenter to select and justify an arbitrary threshold. Second, the algorithm guarantees exclusion of connections that would reasonably be described as superfluous, but third, it simultaneously guarantees that no nodes or node clusters are disconnected from the larger network. Finally, analyses of functional connectivity defined by MCC have been shown to be more sensitive to cognitive load [69].

**Fig 7:**
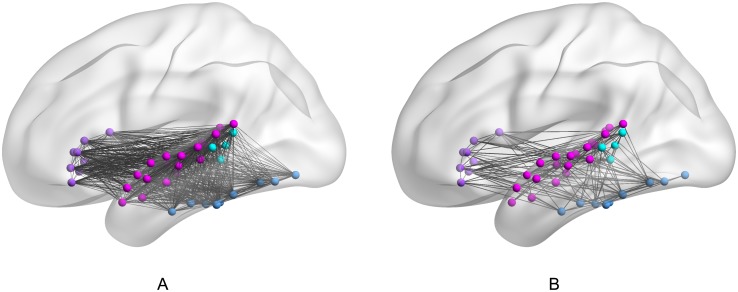
Network connectivity in two individuals with extreme connectivity metric scores.

The Brain Connectivity Toolbox (BCT) [32] was applied to each of the binary connectivity matrices to produce quantifiable metrics of segregation and integration for each participant’s reading network during each of the three modality conditions. We used two measures of segregation (modularity and transitivity) and one measure of integration (global efficiency); the formulas used for these computations can be found in Rubinov and Sporns [32]. We used the default resolution parameter of 1 when calculating modularity. All functions were optimized for binary undirected networks. The segregation and integration measures for each adjacency matrix were averaged for the two runs within each presentation modalities to compute a single average modularity, transitivity, and global efficiency score for each presentation modality and for each participant. Fig 7 depicts the functional connectivity within the 43-node reading networks of two participants: one with low modularity and high transitivity (7A), and the other with high modularity and low transitivity (7B), and highlights the variability among these networks that is critical to our correlation-based analysis.

BCT threshold values equal to 2 standard deviations above or below the mean for each measure were computed, and outlier values beyond these thresholds were replaced with threshold values. A hierarchical linear regression predicted reading skill, as measured by the pseudo-word decoding (PDE) scores obtained during standardized testing, as a function of the BCT measures. Participant age (in months), task accuracy, and task response latency were treated as nuisance regressors and entered in the first step. Segregation and integration measures from each of the three presentation modality conditions were our predictors of interest. Because, as the GLM presentation condition contrasts will show, the three presentation modality conditions were expected to place different demands on auditory and visual processing and integration, and consequently induce different functional connectivity patterns, the BCT measures for the different presentation modalities were entered into the model separately. This strategy allowed us to explore differences in the predictive ability of neural processing dynamics among the task conditions. The question of whether reading difficulty can be explained by functional connectivity patterns within the reading network was addressed by testing whether the BCT measures were significant predictors of PDE scores, once variance attributable to the nuisance regressors has been accounted for. We repeated this hierarchical regression analysis on the significant clusters outside of the core reading network to explore whether our results describe a special property of the reading network. To assess the relative importance of each predictor variable we calculated bootstrap confidence intervals for the differences in contribution to the variance in PDE scores.

## Results

### Behavioral analysis

A one-way analysis of variance (ANOVA) was conducted on accuracy between presentation modalities. Accuracy did not differ depending on presentation modality, *F* (2,46) = 2.43, *p* = .12. The overall participant accuracy was .62 (range = .51-.85). Response latency comparisons between presentation modalities using a one-way ANOVA found response times differed across presentation conditions, *F* (2, 46) = 6.35, *p* = .005. Responses in the AA (*M* = 1615 ms) condition were slower than those in the AV (*M* = 1455 ms; *t* (23) = -3.88, *p* = .001) condition and the VV condition (*M* = 1406 ms; *t* (23) = -2.75, *p* = .011). Response latencies in the AV and VV conditions did not significantly differ *t* (23) = -0.651, *p* = .52. The overall mean response latency was 1493 ms (range = 900–1863 ms).

### Surface-based functional analysis

The sole purpose of the group-level functional analysis was to identify the core left-hemisphere reading network using an unbiased data-driven approach. Because we wished to investigate presentation modality-related differences, we avoided introducing biases in our node selection by collapsing across all runs, thereby restricting the analysis to those regions that were significantly associated with task performance across modality conditions. Table 2 indicates the peak activations within the cluster-size-corrected significant clusters in the left hemisphere (FWE *p* < .05; uncorrected *p* < .001). The significant clusters appearing in the left hemisphere are illustrated in Fig 6. Across all modality presentation conditions, a core left hemisphere network including Fusiform Gyrus, Superior Temporal Gyrus (extending to Posterior Superior Temporal Sulcus) and Inferior Frontal Gyrus showed significantly greater activation during rhyming judgment trials than the baseline fixation response baseline, corresponding to the expected anatomical extents of the core left-hemisphere reading network.

**Table 2:** Significant left hemisphere clusters for the Lexical—Fixation contrast across presentation modalities.

Region	Size (mm^2^)	X	Y	Z	Max t	P
Superior Temporal Gyrus	2632	-61	-13	1	11.58	0.0001
Lingual Gyrus	3946	-6	-68	1	8.81	0.0001
Inferior Frontal Gyrus	6459	-32	32	4	7.87	0.0001
Precentral Gyrus	91	-52	-8	44	7.16	0.0128
Superior Frontal Gyrus	489	-10	15	52	7.12	0.0001
Lateral Occipital Gyrus	281	-23	-87	-12	5.84	0.0001
Fusiform Gyrus	118	-42	-36	-22	5.23	0.0038
Fusiform Gyrus	120	-39	-25	-23	5.21	0.0035
Precentral Gyrus	104	-57	-1	19	4.71	0.0067
Superior Parietal Gyrus	297	-23	-79	18	4.62	0.0001
Lateral Occipital Gyrus	303	-26	-86	3	4.05	0.0001

**Fig 8:**
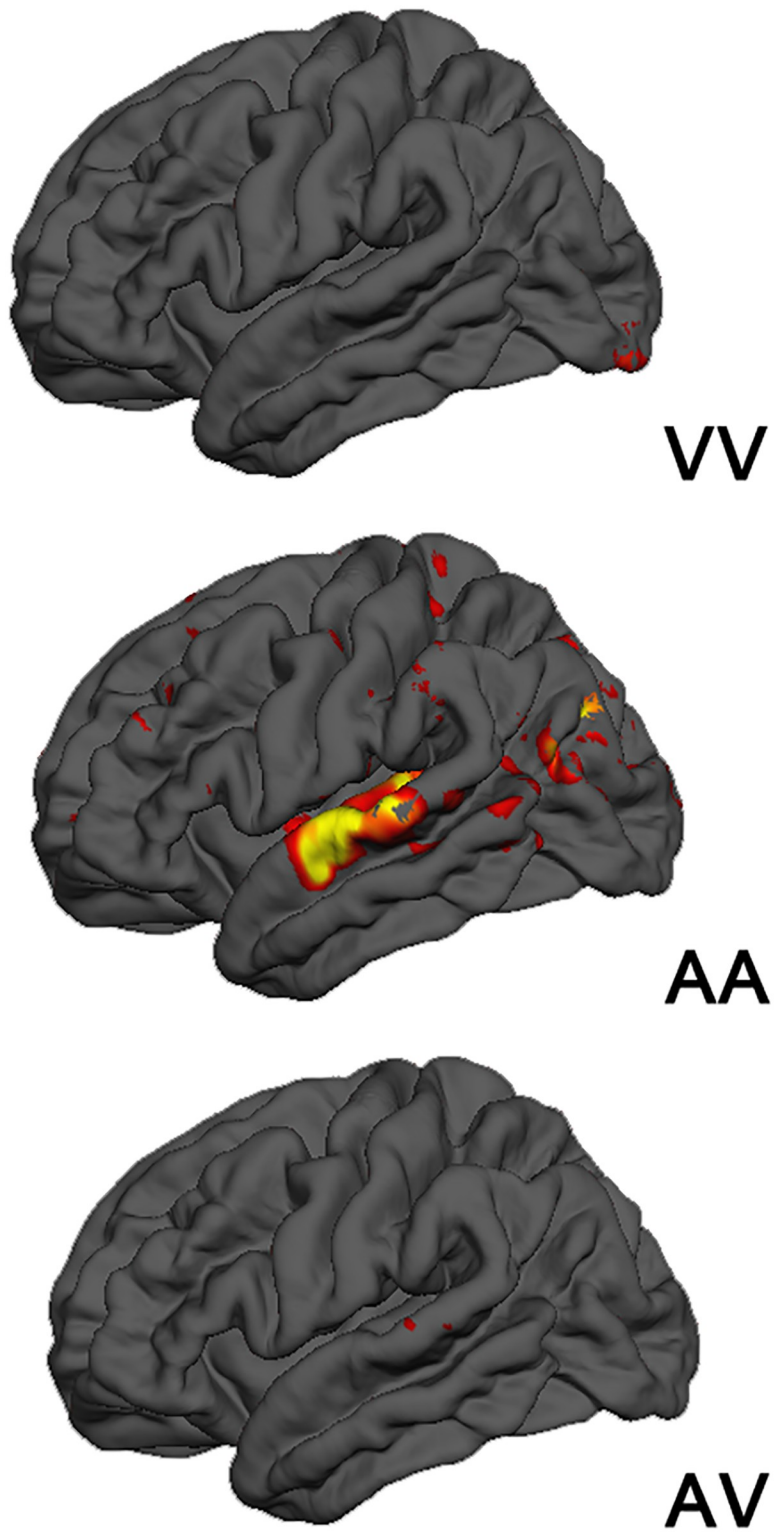
**Group-level contrasts between presentation modality conditions:** [VV vs. AA + AV] (top), [AA vs. AV + VV] (middle), [AV vs. AA + VV] (bottom) using a voxel-wise significance threshold of p = .01 (uncorrected).

Group-level presentation modality contrasts were carried out using a cluster-size corrected significance threshold of *p* < .05, but an uncorrected *p* < .01 threshold to allow for the reduced statistical power arising from partitioning the data into thirds. At these thresholds, only the AA vs other modality contrast generated cluster-size corrected significant activation differences, which we summarize in Table 3. Fig 8, however, shows all clusters meeting the voxel-wise significance threshold, as these patterns conform to the expected contrast pattern: Activation trends in primary and secondary sensory auditory and visual processing regions were commensurate with the reliance of that condition on either vision or audition. STG activation was greater in the AA condition than for the AV and VV conditions. Conversely, though cluster-size significance thresholds were not met, occipital activation was numerically greater for the VV condition than for the AA and VV conditions. As a blending of the two unimodal conditions, though cluster size significance thresholds were not met, the AV condition showed a trend of greater activation than the AA and AV conditions only in a small cluster in the posterior STS. Thus, the interpretation of the network dynamics that follows is done in light of this modality-dependent pattern of sensory processing. As these tasks require different processes (sound matching for AA vs letter to sound mapping then sound matching for VV) it is expected that connectivity would also differ between these conditions.

**Table 3:** Significant left hemisphere clusters for presentation modality contrasts.

Contrast	Region	Size (mm^2^)	X	Y	Z	Max t	P
AA vs Other							
	Superior Temporal	2118	-61	-13	0	7.15	0.0001
	Lingual Gyrus	1017	-31	-47	-6	5.92	0.0001
VV vs Other							
	No significant clusters						
AV vs Other							
	No significant clusters						

### Connectivity analysis

The first-level hierarchical linear regression model including only the nuisance variables did not significantly predict the variance in PDE scores (*F*(3,20) = 1.00, *p* = .41, adjR^2^ = .001). The second-level model that additionally included the BCT measures significantly predicted PDE scores (*F*(12,11) = 2.89, *p* = .04), with an adjusted R^2^ of .50, and predicted significantly more variance in PDE scores compared to the model containing only nuisance variables, *F*(9,11) = 3.19, *p* = .04 (Table 4). Connectivity metrics of the unimodal visual (VV) networks were not predictive of reading ability, however modularity of the crossmodal AV condition and transitivity and global efficiency of the unimodal auditory (AA) condition did predict PDE scores. These results indicate that the network connectivity metrics of network segregation and integration characteristics while engaged in a task that places demands on the orthographic and phonological systems is predictive of reading skill, and suggest that these relationships are dependent on task modality.

**Table 4:** Hierarchical linear regression analysis for the reading network defined using a significance-based threshold (p < .05).

	*B*	*SE*	*β*	*η*^*2*^
(Constant)	-790.65	232.01		
Age	0.21	0.13	0.46	.43
Response Latency	0.02	0.01	0.48	.58*
Accuracy	-14.91	27.31	-0.15	-.16
VV Modularity	115.39	119.65	0.80	.28
AV Modularity	580.64	183.28	4.29	.69 **
AA Modularity	49.55	276.91	0.25	.05
VV Transitivity	-148.70	104.87	-2.54	-.39
AV Transitivity	115.63	118.94	2.09	.28
AA Transitivity	-642.37	184.48	-7.64	-.72 **
VV Global Efficiency	338.06	154.98	3.96	.55
AV Global Efficiency	78.65	175.53	0.92	.13
AA Global Efficiency	1036.82	287.28	8.63	.74 **

Note:

* *p* < .05;

** *p* < .01.

As discussed earlier, tension may exist between connectivity patterns that may work at cross-purposes within a network. In several instances, the regression analysis shows opposite-signed partial correlations involving different connectivity measures. We may ask, for example, whether AA transitivity is more predictive of PDE than is AV transitivity, ignoring the fact that the relationship is negative in one case, and positive in the other. A statistical test of whether opposite-signed correlations differ from each other is not particularly informative, first, because this difference will necessarily be significant when at least one of the correlations are significant, but second because such a test does not answer whether either measure is a more important predictor of reading skill, ignoring the direction of the correlation. The relative importance of the BCT measures within- and across modalities was tested using the relaimpo package in R [70], which assesses relative importance of regressors in the linear model. Contrary to our hypothesis, the predictive relevance of the presentation modality conditions did not partition into unimodal versus cross-modal networks. Rather, as Table 5 indicates, the partial correlations involving each presentation modality within each connectivity metric were equally important predictors of reading difficulty between conditions, even for those measures for which one predictor is significant and the other is not. Additionally, the orthogonal analysis found connectivity metrics are equally important predictors of PDE scores within each modality (Table 6). Thus, though connectivity metrics significantly differed between presentation modalities in many instances, it did not appear to be the case that these measures were more or less important for one presentation modality than the others.

**Table 5:** Differences in relative importance of predictors of pseudo-word decoding scores between presentation modalities within connectivity measures.

BCT	Contrast	Difference
Modularity	VV—AV	-0.01
	AV—AA	-0.06
	VV—AA	-0.07
Transitivity	VV—AV	-0.03
	AV—AA	0.05
	VV—AA	0.02
Global Efficiency	VV—AV	-0.03
	AV—AA	-0.05
	VV—AA	-0.08

***Note***: No contrasts were significant at *p* < .05.

**Table 6:** Differences in relative importance of predictors of pseudo-word decoding scores between connectivity measures within presentation modalities.

Modality	Contrast	Difference
VV	Modularity—Transitivity	0.01
	Transitivity—Global Efficiency	- 0.01
	Modularity—Global Efficiency	0.01
AV	Modularity—Transitivity	-0.02
	Transitivity—Global Efficiency	0.00
	Modularity—Global Efficiency	-0.01
AA	Modularity—Transitivity	0.00
	Transitivity—Global Efficiency	-0.00
	Modularity—Global Efficiency	0.00

***Note***: No contrasts were significant at *p* < .05.

The above analyses indicate that metrics quantifying the topographical organization of the core reading network predicts reading skill. To assess whether this property is unique to the core reading network, we repeated this analysis for the 154-node network constructed using the same methodology from significant clusters outside of the core reading network. The first-level hierarchical linear regression model including only the nuisance variables did not significantly predict the variance in PDE scores (*F*(3,20) = 1.00, *p* = .41, adjR^2^ = .001). The second-level model that additionally included the BCT measures also did not significantly predict PDE scores (*F*(12,11) = 0.78, *p* = .66), with an adjusted R^2^ of -.13, and did not predict significantly more variance in PDE scores compared to the model containing only nuisance variables, *F*(9,11) = 0.75, *p* = .66. This suggests that the relationship between network organization and reading skill in children with reading difficulty is unique to the core reading network.

We repeated the above hierarchical regression analysis on the minimum connected components within the reading network. The results of these analyses, summarized in Table 7, were consistent with those carried out on the networks constructed with a simple significance threshold, and further support the hypothesis that functional connectivity within the core reading network is predictive of reading ability, and that these relationships are sensitive to presentation modality. Within the MCC networks, the first hierarchical linear regression model including only the nuisance variables did not predict reading ability (*F*(3,20) = 1.00, *p* = .41, adjR^2^ = .001). The second level of the model that additionally included the BCT measures did significantly predict PDE scores (*F*(12,11) = 3.54, *p* = .02) with an adjusted R^2^ of .57, and predicted significantly more variance in PDE scores than did the first level model, *F*(9,11) = 3.95, *p* = .02.

**Table 7:** Hierarchical linear regression analysis for the reading network defined using the minimum connected component.

	*B*	*SE*	*β*	*η*^*2*^
(Constant)	50.15	39.22		
Age	-0.05	0.12	-0.12	-.13
Response Latency	0.01	0.01	0.20	.31
Accuracy	35.11	23.25	0.36	.41
VV Modularity	-13.08	23.71	-0.28	-.16
AV Modularity	-41.93	41.46	-0.55	-.29
AA Modularity	19.82	43.34	0.29	.14
VV Transitivity	-61.87	18.77	-1.10	-.71**
AV Transitivity	75.78	23.62	1.04	.70**
AA Transitivity	-36.84	24.20	-0.48	-.42
VV Global Efficiency	28.77	29.94	0.58	.28
AV Global Efficiency	-101.72	32.23	-1.29	-.70**
AA Global Efficiency	30.48	33.43	0.50	.27

Note:

** *p* < .01.

Consistent with the previous analysis, the relationship between functional connectivity and reading abilities appears to be unique to the MCC of the core reading network, and does not extend to the MCC spanning activated regions outside the core reading network. The first level of the hierarchical linear regression only containing nuisance regressors did not predict PDE scores (*F*(3,20) = 1.00, *p* = .41, adjR^2^ = .001). The second level of the model additionally including the BCT measures of the MCC of the regions outside the core reading network did not significantly predict PDE scores (*F*(12,11) = .51, *p* = .87) with an adjusted R^2^ of -.34, and did not predict significantly more variability in PDE scores than did the first level of the model, *F*(9,11) = 0.43, *p* = .89.

## Discussion

We investigated whether task-dependent graph-theoretic measures that quantify the topography of the global reading network predict reading skill in children with reading difficulty. Differences in pseudo-word decoding efficiency scores were predicted by the connectivity measures of the functionally-defined reading network in our sample, but were not predicted by connectivity among regions outside the core reading network. Moreover, this finding was replicated using two very different approaches for network construction. This indicates that reading difficulty can be explained, at least in part, by the characteristic segregation and integration patterns of the reading network under different presentation modality conditions. Network segregation and integration measures showed a modality-dependent relationship with reading difficulty, presented in Table 4 and summarized in Table 8 below. Measures of segregation and integration appeared to be equally important predictors of variance in pseudo-word decoding (PDE) scores, which may be a consequence of the tension between these two measures which are balanced in so-called small-world networks that are commonly associated with optimal network processing dynamics [71]. Interestingly, the regression analyses showed that the crossmodal AV condition generally patterned differently than the unimodal AA and VV conditions: In four of the five cases where at least one metric was a significant predictor of PDE, the AV condition either was the only significant predictor, or else was in the opposite direction (and therefore significantly different from) the unimodal condition. This pattern is consistent with the hypothesis that task conditions that emphasize audiovisual processing are particularly sensitive to reading skill in children with reading difficulty.

**Table 8:** Summary of valences of modality dependent correlations between connectivity metrics and reading skill.

	*Network Segregation*				*Network Integration*	
	*Modularity*		*Transitivity*		*Global Efficiency*	
	Sig	MCC	Sig	MCC	Sig	MCC
**VV**	+	-	-	-*	+	+
**AV**	+*	-	+	+*	+	-*
**AA**	+	+	-*	-	+*	**+**

Note:

* denotes statistically significant relationships. Sig.: Significance-based connectivity threshold; MCC: Minimum Connected Component connectivity threshold

### Measures of network segregation

When examining the statistically thresholded networks, we found that PDE scores were higher for those whose reading network was more modular across all conditions, but the relationship was significant only in the cross-modal condition (AV). Broadly speaking, a network with high modularity has multiple processing clusters (or modules) that function with relative independence from one another [33]. Although these modules are necessarily situated among brain regions that have been traditionally associated with specific functions supporting reading, it is not necessarily the case that these modules conform to the anatomical bounds of these regions. Lower modularity of a network may indicate that the phonological processing supporting these rhyming judgments occurred with relatively little coordination with orthographic processing regions for those with more severe reading difficulty, and that this effect is most pronounced in the audiovisual condition. This is consistent with previous research showing an audiovisual disconnect in dyslexic children [11]. The unimodal AA and VV conditions may have been less likely to promote interactions among phonological and orthographic processes because these presentation conditions did not guarantee them: Phonological judgments on auditory-only input can be accurately made without orthographic input, and these judgments on visual-only input may have relied on orthographic cues, such as orthographic overlap. Overall, this suggests that children with comparatively mild reading impairment demonstrate greater segregation (i.e., less communication between local network circuits) to accommodate the audiovisual integration processes that support more fluent reading through more effective mapping between orthography and phonology, and lends further support to the argument that an impairment of audiovisual integration processes contributes critically towards reading difficulty [11, 72].

Modularity among MCC-thresholded networks did not appear to be significantly predictive of PDE, and presentation modality conditions did not pattern in the same way as the statistically-thresholded networks. Modularity scores were higher (*M* = .29) and much more variable (*SD* = .13) for the MCC networks than for statistically thresholded networks (*M* = .04, *SD* = .03), and so the predictive strength of the modularity of statistically-thresholded networks may have been driven by extreme scores.

Transitivity within the network under different modality conditions—that is, the propensity for the network to form recurrent connectivity loops—was higher for poorer readers when both words were presented in the same modality (AA and VV), but higher for better readers in the crossmodal presentation condition (AV). This pattern was consistent for both thresholding techniques, but significant only for AA among significance threshold networks, and for VV and AV among MCC networks, which may indicate that this relationship is driven by recurrent loops formed by the strongest functional connections. Though transitivity among functional connectivity networks has not been previously examined as a factor in reading ability, from a network processing perspective, recurrent connections permit previous neural computations to influence subsequent processing. Such an architecture potentially supports a memory circuit, and relatedly, error checking using informative prior knowledge [73]. If we interpret transitivity as a marker of processing uncertainty, this pattern may indicate that higher transitivity in poorer readers in the unimodal conditions reflect an inability to quickly settle on a stable orthographic or phonological pattern in these participants under unimodal presentation conditions—i.e., difficulty maintaining clear phonological and orthographic representations is a marker of especially poor reading ability. Conversely, children who are better at mapping between orthography and phonology do so with less recurrent processing under crossmodal presentation conditions, and efficient crossmodal mapping may be a sign that a child is overcoming reading difficulty. It is important to note that although network transitivity relates to reading skills, we do not know where these recurrent connections exist within the network because these measures characterize connectivity among all nodes throughout the network. It is possible that these recurrent loops exist within discrete modules or between modules, for example, reflecting mutual isolation of the orthographic and phonological networks. Unfortunately, the global network transitivity measure does not permit conclusions at this level of specificity, and so further research, perhaps using a seed-based approach, is needed to examine the role of local transitivity in reading difficulty.

### Measures of network integration

Increases in global efficiency—fewer links—were associated with better PDE scores when both words were presented audibly (AA). We note that the other conditions (AV,VV) showed a similar, but non-significant, trend, and thus this relationship suggests overall reading network efficiency is related to higher PDE scores. We interpret this pattern in light of the phonological nature of the rhyming judgment task, which requires encoding visually-presented words into a phonological form. Again, the global network analysis cannot determine the frequency and conditions under which individual connections were used, however this presentation-dependent relationship indicates that the children with the least reading difficulty were most likely to recruit less of the network’s circuitry. Given the phonological nature of the task, this network likely includes regions involved in resolving letter-sound correspondences during audiovisual integration. If reading difficulty is monotonically related to disordered audiovisual integration processing, involvement of these audiovisual integration sites may be more helpful for better readers. If audiovisual processing is disordered in these individuals, input from integration sites would be expected to hinder performance, and children with less severe reading difficulty may be those who better gate orthographic input into the phonological system. When words were presented audibly, participants had direct access to accurate and robust phonological representations, and accurate performance is less reliant on audiovisual integration processes. This interpretation is supported by the GLM presentation modality contrasts showing an extensive region of STG in which activation in the AA condition was significantly greater than that for the other two conditions, and possibly reflecting a more efficient recruitment of the local phonological network. The manner in which information is gated into the visual system has been proposed as a contributing factor of reading difficulty [74, 75]. It seems equally plausible that reading might also depend on appropriate gating of information into the phonological system.

Analyses of global efficiency within MCC networks found the unimodal conditions maintained the same directionality, but found that global efficiency was the only significant and negatively related predictor of PDE. Mean global efficiency was lower overall in the MCC networks (M = .55) than the significance-thresholded networks (M = .92), indicating an increased sparsity of these networks. This suggests that it is the strongest connections among networks in the AV presentation condition that drive this relationship, again highlighting the sensitivity of tasks emphasizing audiovisual processing to reading difficulty.

### Considerations and limitations

Our analyses were constrained or influenced by features of our data set that provide important context for the interpretation of our results. First, the fast event-related design used in the study was optimized for a more conventional voxel-based exploration of task-dependent regional processing in this population under different conditions using the General Linear Model. As we suggest earlier, the onsets of trials of different types would plausibly influence the computation of lag-dependent cross-correlations, thus motivating the decision to focus on zero-lag correlations. Though a block design would appear to overcome this obstacle by permitting computation of connectivity within-blocks, such a design would make the task trivially easy and was thus precluded.

Second, we applied two of the many thresholding techniques available. Both techniques lead to the same general conclusion that reading difficulty is predicted by task-related functional connectivity specific to the reading network, and that these relationships are dependent on audiovisual processing modality, however subtle differences were found between them. We note that graph theoretic measures depend on the manner in which network topologies are defined [68], and thus that a different thresholding technique can generate different measures of network integration and segregation. Thus, that differences between approaches were observed was not surprising, but moving beyond our speculative discussion of these differences to uncover the theoretical implications and mechanisms underlying these differences requires further investigation that we would encourage.

## Conclusions

A large body of literature supports the intuitive argument that reading is dependent on strong connections between functionally specialized regions within a larger reading network supporting orthographic and phonological processing [20, 36–42, 44–49, 53, 54]. Much of the research that has been conducted has focused primarily on the strength of connectivity between specific brain regions [50–54]. Our findings complement this literature, showing that connectivity strength is only one consideration, and that the manner of connectivity—whether to facilitate communication between or within regions, or to promote recurrent information processing—also plays an important role in reading skill. We show that the manner of functional connectivity throughout the entire reading network is predictive of reading skill in children with reading difficulty. This finding is consistent with the assumption that the manner in which these putatively functionally-specialized regions are connected impacts how they interact during reading and thus influences reading ability in children with reading difficulty.

Though no presentation modality was clearly more sensitive to network structure, we do find that children with reading difficulty engage the left hemisphere reading network differently depending on presentation modality and reading skill, as shown by the linear regression coefficient valence differences across presentation modality conditions. Thus, we found presentation-modality dependent processing dynamics differentiate better from poorer readers with reading difficulty. Our results suggest that children better able at overcoming reading difficulty do so by balancing increased network modularity with processing efficiency, and that this advantage is more pronounced under conditions that emphasize audiovisual processing.

Our findings indicate that characteristic patterns of segregation and integration within the reading network predicts severity of reading difficulty in children diagnosed with developmental reading disorders, and thus establishes a relationship between reading difficulty and the manner in which information is transferred through the network of brain areas involved in reading. Moreover, these relationships varied by presentation modality, suggesting that a child’s ability to modulate network processing dynamics in response to different task-modality demands may be an important factor in reading skill. Complementing conventional contrast-based analyses, these functional connectivity analyses of reading provide important additional insight into the processing dynamics that may underlie reading difficulty.

## Supporting information

S1 File GZIP compressed time series and individual differences regressor data files. Detailed information about the archive contents is provided in the included README.txt file.
(TGZ)
